# Body Art: A Study on Aesthetics and Postural Instability in Patients With Piercings

**DOI:** 10.7759/cureus.87017

**Published:** 2025-06-30

**Authors:** Fabrizio Caricato, Anna Maria Noto, Eleonora Sanzone, Paola Donati, Paolo Domenico Parchi

**Affiliations:** 1 Dentistry, Independent Researcher, Rome, ITA; 2 Clinical Kinesiology, Independent Researcher, Siracusa, ITA; 3 Orthopedics, Independent Researcher, Brescia, ITA; 4 Orthopedics and Trauma Surgery, University of Pisa, Pisa, ITA

**Keywords:** body art, body posture, cranial nerves (cn), piercing, postural dysfunction syndromes (pds), posture-dependent symptoms, posture variations, proprioceptive system, trigeminal nerve stimulation

## Abstract

Body art is increasing in popularity around the world and may now be considered a mainstream activity even in Western society. In fact, nowadays, this practice has become customary, independent of social and cultural origin. The key motivating factors for obtaining body piercings are individual expression, personal image management, and art. Research in psychology and sociology has shown that the body is not considered acceptable if it is not covered with signs of belonging; that is the reason why an increasing number of people adorn themselves with often permanent decorations. As the prevalence of body art has increased, adverse health risks associated with body piercing have also been documented.

In this work, after taking into consideration how piercings stimulate certain nerve endings and having specifically evaluated the areas pertaining to the trigeminal nerve and those related to the hypoglossal nerve, we carried out a clinical study on 26 patients according to the Integrated Postural Analysis (API) method by the Italian Association for Postural Occlusal Re-education (AIROP) on how facial piercings can influence the individual's posture.

These evaluations led us to consider how the systems involved, postural and proprioceptive, can respond more or less intensely to the stimuli induced by piercings and that these cause alterations; furthermore, the responses to such solicitation can vary based on the onset and integration of the stimulus itself and the compensatory capacity of the neurophysiological system.

We can thus affirm the existence of a well-founded correlation between postural alterations and the presence or removal of piercings, which can lead to manifestations referable to postural dysfunction syndromes (PDS).

## Introduction

The topic of interest of this study arises from the observation of the global diffusion of the form of art that is now defined as “body art,” a term that refers to all procedures, such as tattoos, incisions, and perforations, that use the body as a medium of expression [1]. In fact, nowadays, this practice has become customary, independent of social and cultural origin.

Personal identity refers to the unique ways that people define themselves as individuals. Personal identity markers are often the things we choose to define ourselves by, such as our appearance, social affiliations, and cultural background. Researches in psychology and sociology have shown that the body is not considered acceptable if it is not covered with signs of belonging: piercings and tattoos, manifestations of body art, have taken on in this context the tacit meaning of rewriting one’s own world even on one’s own body, serving both as an introverted, private and extroverted, public statement toward society [2].

Body piercing and tattoos have increased tremendously in popularity all over the world in recent years and are practiced across many social and age groups, independently of cultural extraction. However, no exact statistics for these practices are available, and estimates of incidence have been derived from studies with few participants, ranging from 6.5% to 56% for subjects with piercings and from 4.5% to 24% for those with tattoos [3,4].

As the prevalence of body art has increased, adverse health risks associated with body piercing have been documented, and healthcare professional needs to be aware of the complications that can occur. Earlobes and ear cartilage are the sites most frequently pierced. Other body parts that are often pierced include eyebrows, noses, cheeks, lips, frenula, tongues, uvulas, nipples, navels, and various genital sites. Minor local complications, including infection, allergies, and various skin, cartilage, or dental problems, occur frequently; also, major systemic complications have been reported, including headaches and back pain, which lead to having to consider body art from a public health perspective as well [5-7].

In this work, after taking into consideration how piercings involve certain nerve endings and specifically evaluating the areas pertaining to the trigeminal nerve and those to the hypoglossal nerve, we applied the Integrated Postural Analysis (Analisi Posturale Integrata) (API) method by the Italian Association for Postural Occlusal Re-education (AIROP) to assess how piercings placed in various points of the head can influence the posture of the individuals examined, conditioning their postural system and basic proprioception [8].

## Materials and methods

**Figure 1:**
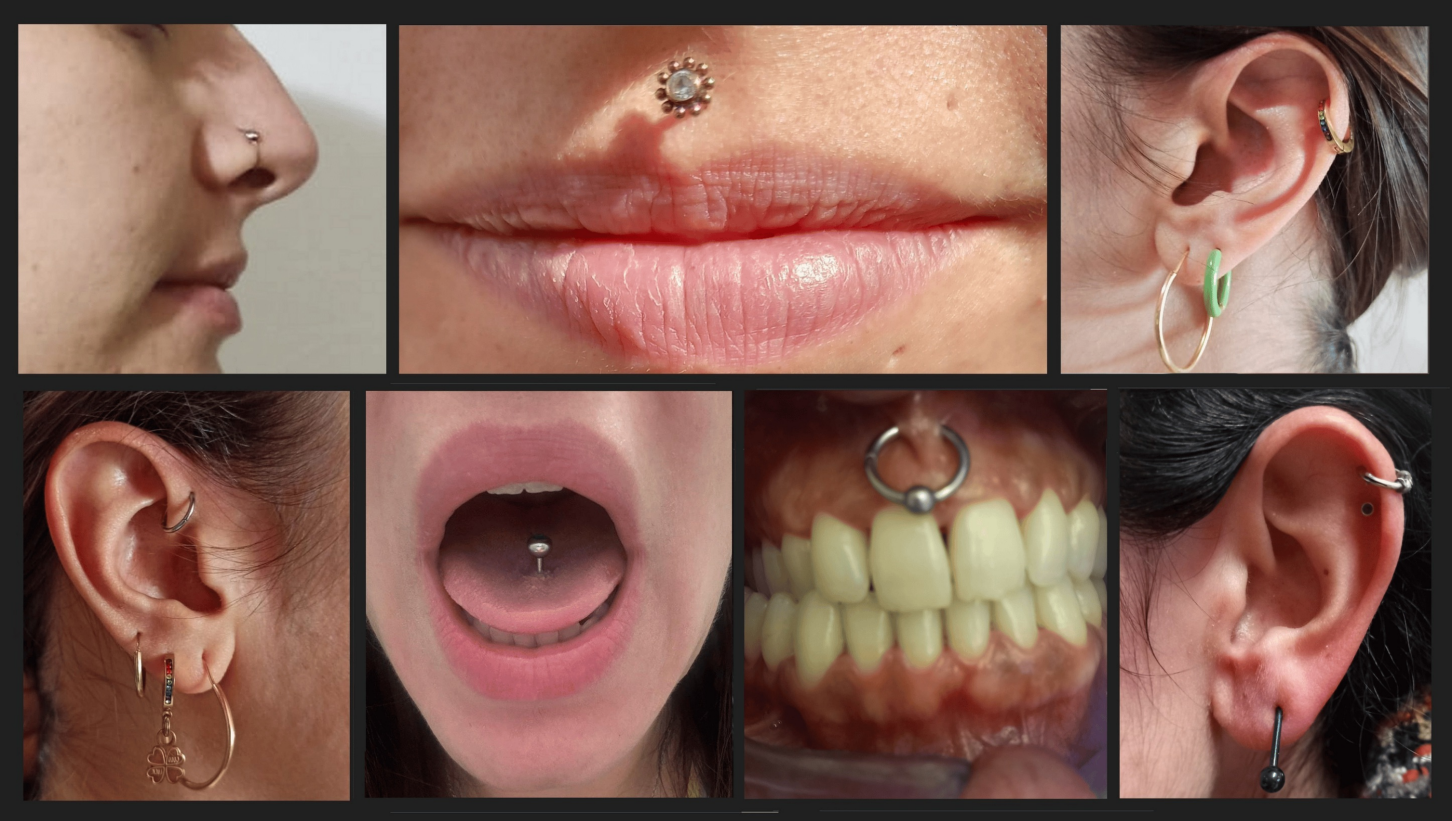
Examples of piercings (personal clinical cases)

In this clinical study, 26 patients aged between 21 and 41 years were examined, who had one or more oral or facial piercings, to focus on piercings pertaining to cranial nerves. Piercings to the auricle, tragus, nose, and tongue were considered, while those to the navel, nipple, and all the rest of the body were excluded (Figure 1). The time elapsed from piercing insertion ranged from three months to 18 years.

Written consent was waived for all study participants. Each patient also underwent a small anamnestic survey to assess lifestyle, medical history, health status, bone trauma or surgery, and presence or absence of pain. A questionnaire specific to the piercing (motivating factors for undergoing body piercing, the area where it is located, and the presence of pain during its application or in the following weeks) was also administered to the patients. The Internal Review Board of the University of Pisa pre-approved the study protocol.

All participants were asked to remove their piercings at least 24-48 hours before the application of the API method, to reset the system, and so that postural alterations with and without piercings could be assessed through the tests. A limitation of this study is that the participants did not agree to a longer time interval between the phase 1 and phase 2 tests; if a longer time had elapsed, the holes of the piercing would have closed, at least partially, hindering their relocation.

Postural tests performed

According to the API method by AIROP, the tests that we have performed for this study are as follows: the postural Romberg test, the postural tone harmony (ATP) test, the Barrè vertical test in antero-posterior (AP) projection, and the index finger test, as well as stabilometry using a baropodometric platform.

Postural Romberg Test

**Figure 2:**
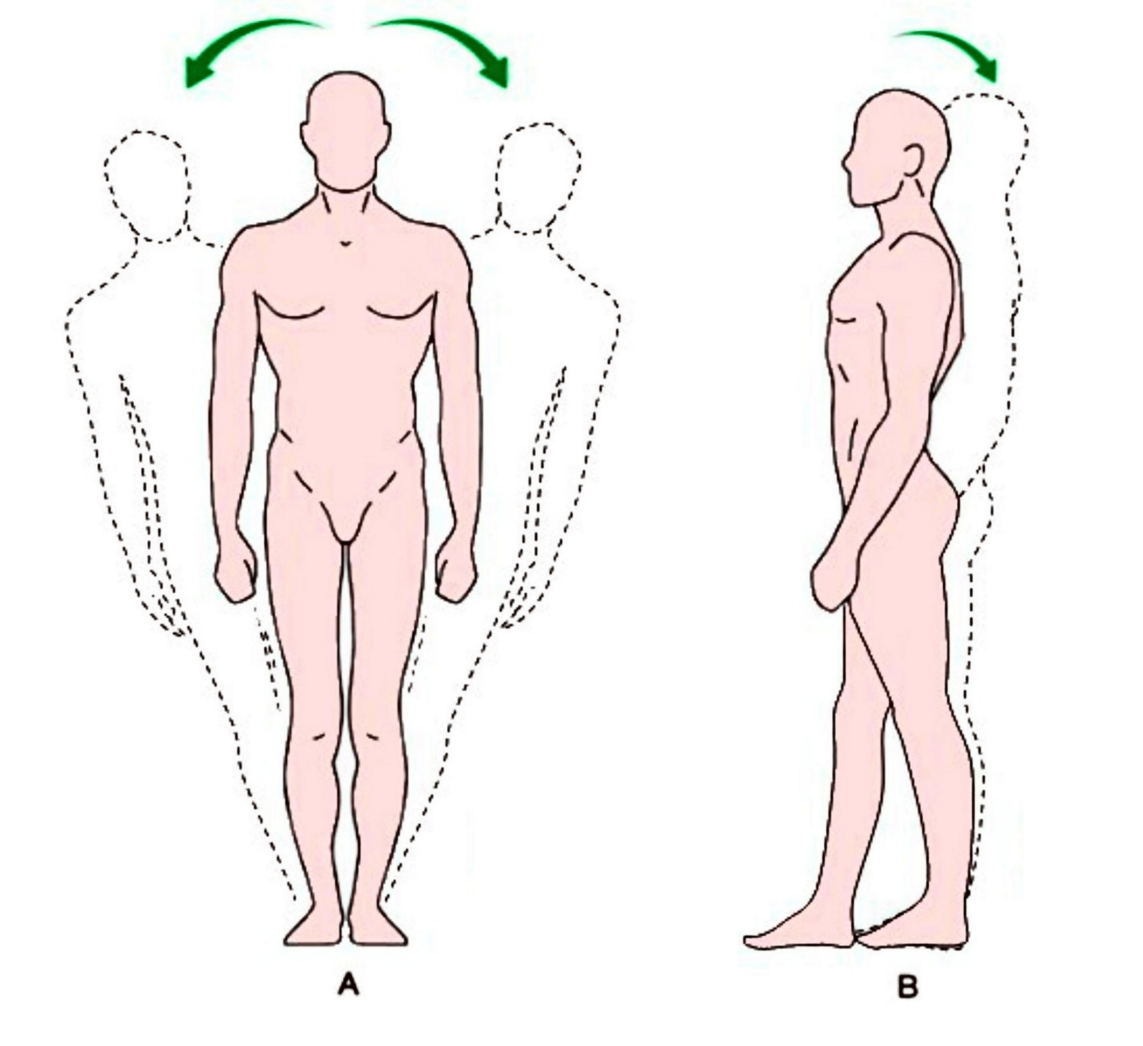
Romberg test The test is negative when the oscillations are maintained by the patient without drifting or falling. The test is positive when the patient cannot maintain balance and lower limb compensation tactics are required. A: latero-lateral oscillations, B: antero-posterior oscillations

The postural Romberg test is a diagnostic test used on patients to evaluate the static otolithic component of the vestibular system; a positive Romberg test indicates a malfunction in one of the systems that maintain balance (Figure 2).

In order to maintain balance, the body uses three main sensory processes: proprioception (the position of the body in space), vestibular function (recognizing the position of the head in space), and vision (perception and disposition of the body with respect to space). The Romberg test is positive when the visual system is canceled by closing the eyes, and the patient is unable to maintain balance.

During the test, the subjects are in an orthostatic position, with feet at 30°, heels together, no muscle tension, and arms pointing forward. We ask the patients to close their eyes, and we wait 20-30 seconds: no abrupt lateral or antero-posterior drifts or falls of the subject should be observed.

**Figure 3:**
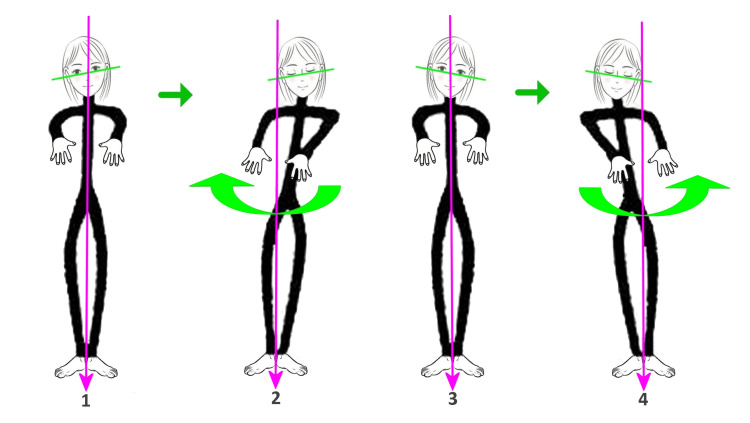
Postural Romberg test 1: Basic condition, with bipupillary line inclined to the right. 2: Physiological rotation to the right and translation to the left. 3: Basic condition, with bipupillary line inclined to the left. 4: Physiological rotation to the left and translation to the right.

It is also necessary to pay attention, before performing the test, to the inclination of the bipupillary axis, bearing in mind that it is never horizontal, but inclined from 1° to 4°, in 70% of cases downward to the right. Under physiological conditions, there will be a slow lateral drift of the patient’s arms. In the case of a bipupillary line inclined to the right, it is physiological to find a rotation on the same side of the inclination (magnified by the extension of the arms), to the right, and a translation of the body (pelvis and shoulders) on the other side of the inclination, therefore to the left; in the case of a bipupillary line inclined to the left, it is physiological to find a rotation on the same side of the inclination (magnified by the extension of the arms), to the left, and a translation of the body (pelvis and shoulders) on the other side of the inclination, therefore to the right (Figure 3).

ATP Test

**Figure 4:**
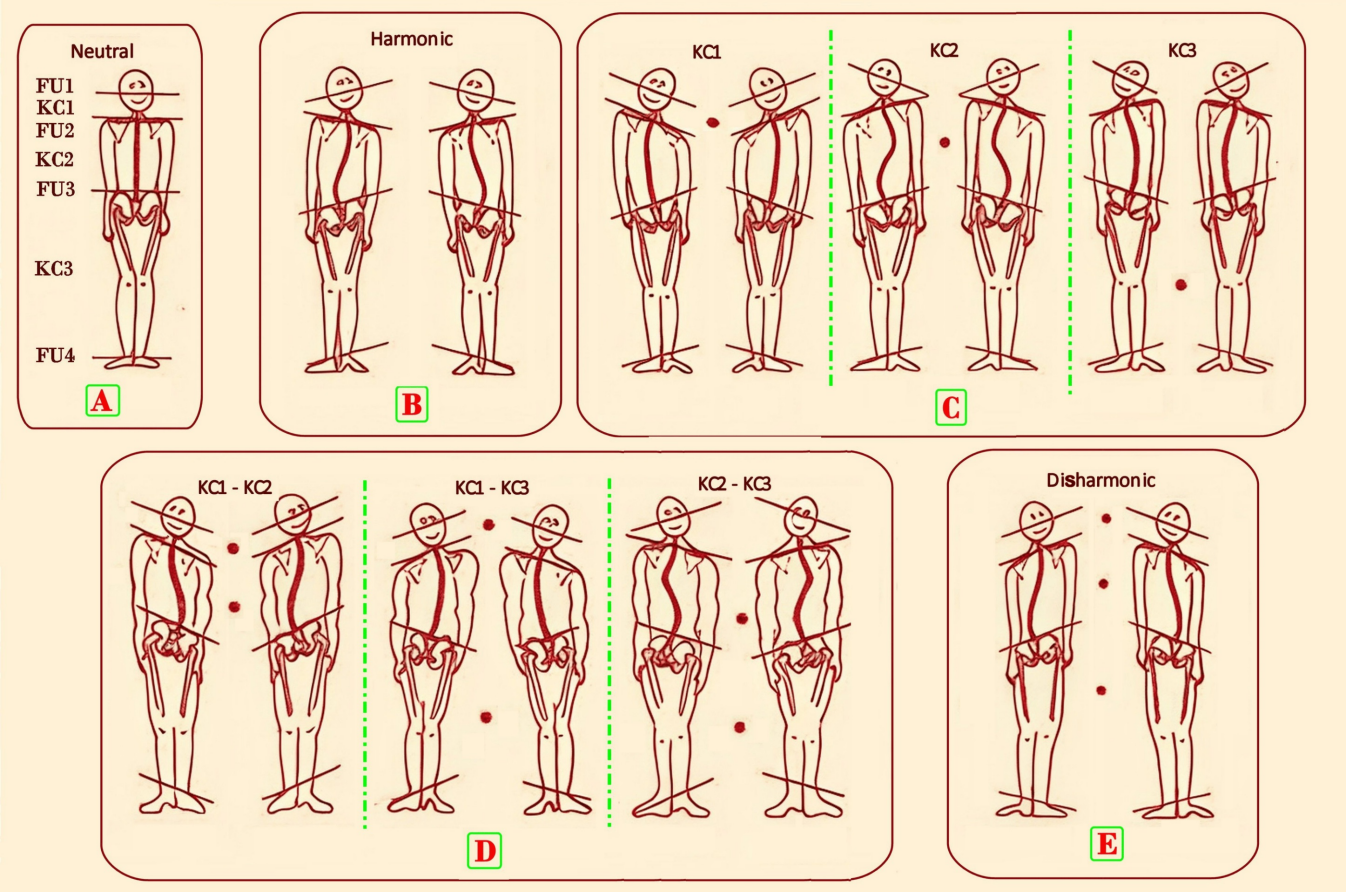
Assessing the harmony of postural tone with the ATP test A: Neutral condition. FU1: Jaw and skull, held together by the TMJ. For the assessment of the postural tone, the occlusal plane and the relative position of both the right and left TMJ must be taken as a reference point. KC1: Occiput and cervical spine. FU2: Scapular girdle, formed by acromion-clavicular, sterno-clavicular, and scapulo-humeral. A line is drawn connecting the two acromion-clavicular joints. KC2: Thoracolumbosacral spine. FU3: Pelvic girdle, sacro-coccygeal, sacro-iliac, and ileo-femoral joints. A line is drawn on the chart by identifying the right and left ASIS through palpation. KC3: Thigh-leg tract. FU4: The foot-ankle complex with the peroneal-tibia-astragalic, sub-astragalic, and calcaneal-cuboid joint. For this unit, the reference is the intermalleolar line, and the parallelism (or not) with the floor plane can also be verified with the data recorded on the podiatoscope examination, by placing the line more cranial on the side where a footprint tending toward the hollow is found. B: Harmonic condition: the four FUs are in a state of dynamic equilibrium. C: Disharmony in one body district, KC1, KC2, or KC3. D: Disharmony in two body districts, KC1-KC2, KC1-KC3, or KC2-KC3. E: Disharmony in all body districts, KC1, KC2, and KC3. ATP: postural tone harmony, TMJ: temporomandibular joint, ASIS: anterior superior iliac spine, FU: functional unit Freely taken and edited with permission from Dr. Zavarella [9].

The ATP test assesses the harmony of postural tone by observing the functional units (FUs) and the kinematic chains (KCs) connecting them [9]. In fact, four functional units (FUs) can be identified in the human body, which are in a state of dynamic equilibrium via the connecting segments of the kinematic chains (KCs) [8,9]. In the ATP test, we must draw a line for each FU on the postural chart and then assess whether or not it is parallel to the horizon and the other FUs. The postural tone is considered harmonic when FU1 and FU2 are convergent (KC1 area), FU2 and FU3 are divergent (KC2 area), and FU3 and FU4 are parallel (KC3 area); otherwise, it is disharmonic (Figure 4).

The joints that constitute the four FUs perform movements in the three planes of space, developing a sort of “buffer system” to postural asymmetries. The reciprocal spatial relationships between the functional units are always assessed by taking into account that physiologically, the first units establish a harmonic relationship in disparallelism (KC1-KC2), while for the third and fourth units, the opposite occurs (KC3). When the relationships of parallelism or disparallelism between the postural units are not physiologically respected, a disharmonic syndrome occurs. Therefore, by identifying the disharmonic syndrome in the relative FU or KC, a structural primacy is detected, and the “buffer” capacity for functional compensation is exhausted. This will be the area where treatment protocols should be targeted to restore the lost compensatory ability [9].

Barrè Vertical Test

The Barrè vertical test in antero-posterior (A-P) projection is a particular type of postural test performed using a digital level or a wire with a small lead weight hanging from it. The test is executed by asking the patient to stay in an upright position and placing the plumb line exactly on the posterior longitudinal line (the one that goes from the cranial vertex to the base of the feet). The operator stands behind the patient and evaluates how the posture landmarks (the vertex V, the body of the seventh cervical vertebra C7, the body of the third lumbar vertebra L3, the intergluteal line, and the projection of this half-line on the ground) are organized in relation to the plumb line. By observing the patient and the landmarks in relation to the plumb line, it is possible to define four postural typologies: ascending syndrome, descending syndrome, mixed syndrome, and whiplash.

Index Finger Test

The index finger test is a neurophysiological test that uses variations in the symmetry of muscle tone determined by internal variations such as spinal tension, podalic support, or variations in cranio-mandibular relations [10,11]. With this test, the reflexes corresponding to the evoked movement are encoded and made manifest through the extension or non-extension of the upper limbs and, in particular, the “index fingers.”

The test is performed by asking the subject to extend their upper limbs with the index fingers forward, without looking at them, with teeth not in contact, and neutral feet. Under normal conditions, we can see the aligned position of the index fingers, or one of them can be advanced. Using this baseline test under physiological conditions as a point of reference, we can evoke different reflexes and note changes in the extension of the indices that will indicate to the therapist where to pay more attention.

**Figure 5:**
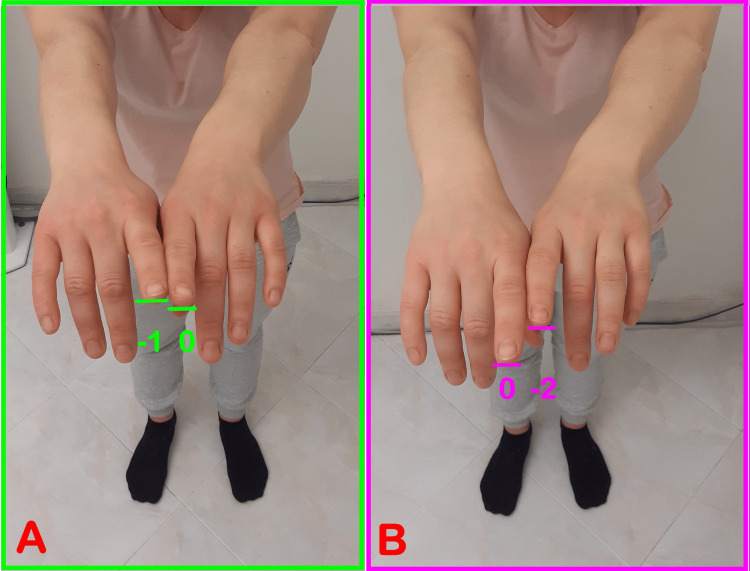
Example of index test interrogation A: Baseline condition. The right limb is always indicated first, setting the advanced index to “0”. The value of the retracted limb will be negative on a scale from 1 to 3. In this case, it results in “-1, 0”. B: Protrusion of the tongue to the left as an interrogation. In this case, it results in “0, -2”, so we found a pathological reflex of the “tongue body district” as the extensor tone on the side where the reflex is evoked has decreased.

The reflexes evoked are the cervical, vestibular, and oculomotor ones. To assess the various anatomical-physiological districts, the patient is asked to make certain movements or assume different positions: for the cervical spine and hyoid bone, the index fingers are observed while the patient flexes, extends, tilts, and rotates the head; the patient is asked to move their tongue, open their mouth, or clench their teeth to assess the motor unit of the jaw (occlusion and tongue); to evaluate the postural muscles of the eye, the patient will be asked to make certain eye movements; to assess how much the support of a single foot affects posture, the patient is placed in a monopodalic stance (Figure 5).

In all interrogations, in physiological conditions, the extensor tone on the side where the reflex is evoked will remain constant or will be increased, except for the oculomotor interrogation (oculogyric reflex), which behaves in the opposite manner of the cephalogyric one.

Static stabilometry with baropodometric platform

The baropodometric platform is an instrument that uses small sensors (force and movement transducers) placed on the surface of a platform to measure plantar pressures in the static phase and dynamic phase. During the static phase, the baropodometric examination provides quantitative and qualitative information on the plantar support and its anatomy, the plantar pressures, and the centers of pressure; it highlights the presence of postural changes, making it possible to observe the effects of prostheses and orthotics or to highlight whether the presence of a device (piercings, gnathological splints, or visual lenses) alters posture.

In the dynamic phase, the baropodometric examination makes it possible to visualize the support of the foot during the step, the weight shift, the sequence of forces present in the vertical plane, the load times during walking, the contact surfaces, and the speed during each acquired step.

## Results

Clinical study structure

The first test carried out on each subject, in the absence of piercings, is the postural Romberg test, performed to exclude any case of balance disturbances and instability caused by the possible presence of injuries to the vestibular apparatus, cerebellum, and labyrinth. Only one patient out of 26 had a positive Romberg test, so the subject was excluded from this study and advised to consult a specialist.

Subsequently, all the tests mentioned previously were first carried out without the piercings, and then, the patients were asked to reinsert them, to repeat all tests and establish whether or not there had been any change in the results (Table 1).

**Table 1:** Number of subjects for each piercing location Details of the number of patients examined for each piercing site.

	Tongue	Tragus	Nose	Auricle	Septum-tongue-tragus
Number of subjects	4	6	3	10	2

The tests were carried out in the following sequence, first without the piercing and then after repositioning it: Romberg test, ATP test, Barré A-P test, and index finger test (taken as a discriminator for the primary attractor), thus moving from a general analysis to a more particular one, which focused on the proprioceptive system involved. Finally, each subject underwent the static analysis using a stabilometric platform (FreeMed® by Sensor Medica, Rome, Italy), in which the podalic load of both feet was taken as reference data.

A total of 200 measurements were collected, of which 25 were from the ATP test (one test per patient), 25 from the Barrè A-P test (one test per patient), and 150 from the index finger test (six interrogations were made per patient).

In total, there were 72 (36%) variations between time 1 (without piercing) and time 2 (with the piercing relocated), of which 44 (22%) worsened, 25 (12.5%) showed an improvement in the tests, and three (1.5%) remained doubtful (Table 2).

**Table 2:** Overall results Overall results showing the changes observed after the reinsertion of the piercings.

	Changes without/with piercing	Worseness	Improvement	Doubt
Overall results	36%	22%	12.5%	1.5%

At the ATP test (Table 3), seven (28%) patients manifested changes; in particular, four (16%) showed a worsening at piercing relocation, with the involvement of more kinematic chains or a tendency toward disharmony; three (12%) improved, manifesting greater postural harmony. Eighteen patients remained unchanged.

**Table 3:** ATP test results ATP test results showing the changes after piercing relocation, and in detail by piercing site. ATP: postural tone harmony

	Total	Tongue	Tragus	Nose	Auricle
Changed	28%	-	-	-	-
Worsened	16%	-	33.3%	33.3%	10%
Improved	12%	50%	16.7%	-	-

Considering the data more specifically, it can be highlighted that on the ATP test, by relocating the piercing to the tongue, there was an improvement in the postural system in two (50%) cases; at the tragus, there was an improvement in one (16.7%) patient, a worsening in two (33.3%), and no change in three (50%); at the nose, there was a worsening in one (33.3%) case and no change in two (66.7%); and at the auricle, we found no change in nine (90%) patients but a worsening in one (10%).

At the Barrè A-P test, on the other hand, replacing the piercings resulted in an unchanged postural attitude in 20 (80%) cases, an improvement in two (8%), and a worsening in three (12%).

With regard to the index finger test, the most significant interrogation was the one concerning the rotation of the head, with positivity for 14 (56%) patients, and of these, 10 showed a worsening (from physiological to pathological) and four an improvement after replacing the piercing. Concerning oculomotricity, there was a change for eight (32%) subjects, of which five showed a worsening and three an improvement. Analyzing the topography of the piercing location (Table 4), we observed that for the tongue, there was a change in 10/24 (41.7%) interrogations, with a worsening in six patients and an improvement in four; for the tragus, there was a change in 11/36 (30%) interrogations, a worsening in eight and an improvement in three; for the nose, there was a change in 11/18 (61%) interrogations, of which there was worsening in eight inquiries and improvement in three; for the auricle, a change was seen in 35/60 (58.3%) interrogations, of which there was worsening in 24 and improvement in 11. When the piercings were multiple (septum-tongue-tragus), all patients showed a change, one with worsening and one with improvement.

**Table 4:** Index finger test results Index finger test results by piercing location, showing the changes after reinsertion.

	Tongue	Tragus	Nose	Auricle	Septum-tongue-tragus
Changes after reinserting the piercings	41.7%	30%	61%	58.3%	100%

Regarding the evaluation through the stabilometric platform, in the present work, we used the static analysis, in which the subject is asked to stay in an upright position on the platform for 30 seconds, without shoes; the platform sensors are able to highlight every slightest oscillation made by the patient during this time. The test is used to quantify the postural control in an upright position and to evaluate the adaptation mechanisms of the nervous system (sensory, motor, and central) involved in the control of posture and balance. This test can be repeated under different conditions (eyes closed, mouth open, with/without bite, with/without piercing, and with/without visual lenses) to assess the postural changes by stimulating the receptor channels we want to examine.

**Figure 6:**
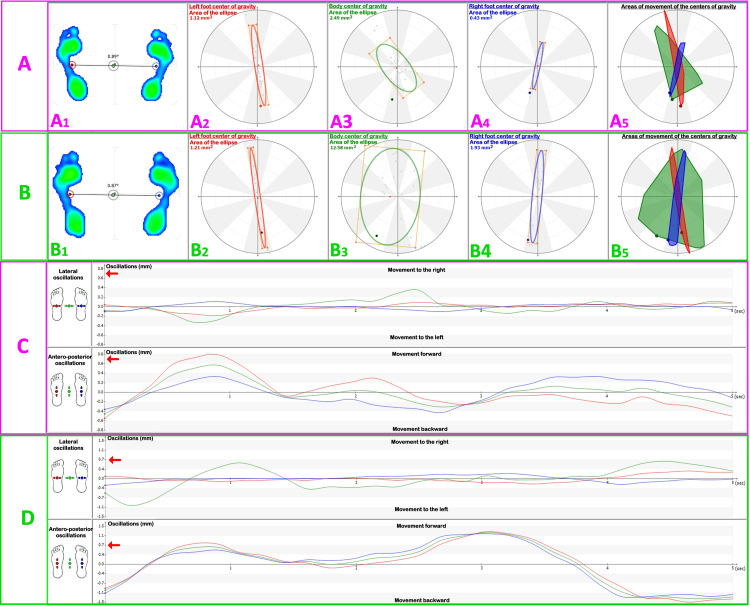
Stabilometric examination results without and with piercing Results of the stabilometric test without and with piercing, carried out on a patient with four piercings in the left earlobe. A: Stabilometric examination without piercing; A1, evidence of the joint segment between the CoFs right and left; A2, area of movements of the left foot center of gravity; A3, area of movements of the body center of gravity; A4, area of movements of the right foot center of gravity; A5, comparison between the three areas of movements of the centers of gravity. B: Stabilometric examination with piercing; B1, evidence of the joint segment between the CoFs right and left; B2, area of movements of the left foot center of gravity; B3, area of movements of the body center of gravity; B4, area of movements of the right foot center of gravity; B5, comparison between the three areas of movements of the centers of gravity. The areas of the oscillations of the body center of gravity are much larger by relocating the piercing. C and D: Comparison on the Cartesian axes of the oscillation graph of the three centers of gravity (blue, right foot; red, left foot; green, body center of gravity). Note the different scale indicated by the red arrows. The oscillations are much wider after relocating the piercing (C: without piercings, D: with piercings). CoFs: center of foot

**Figure 7:**
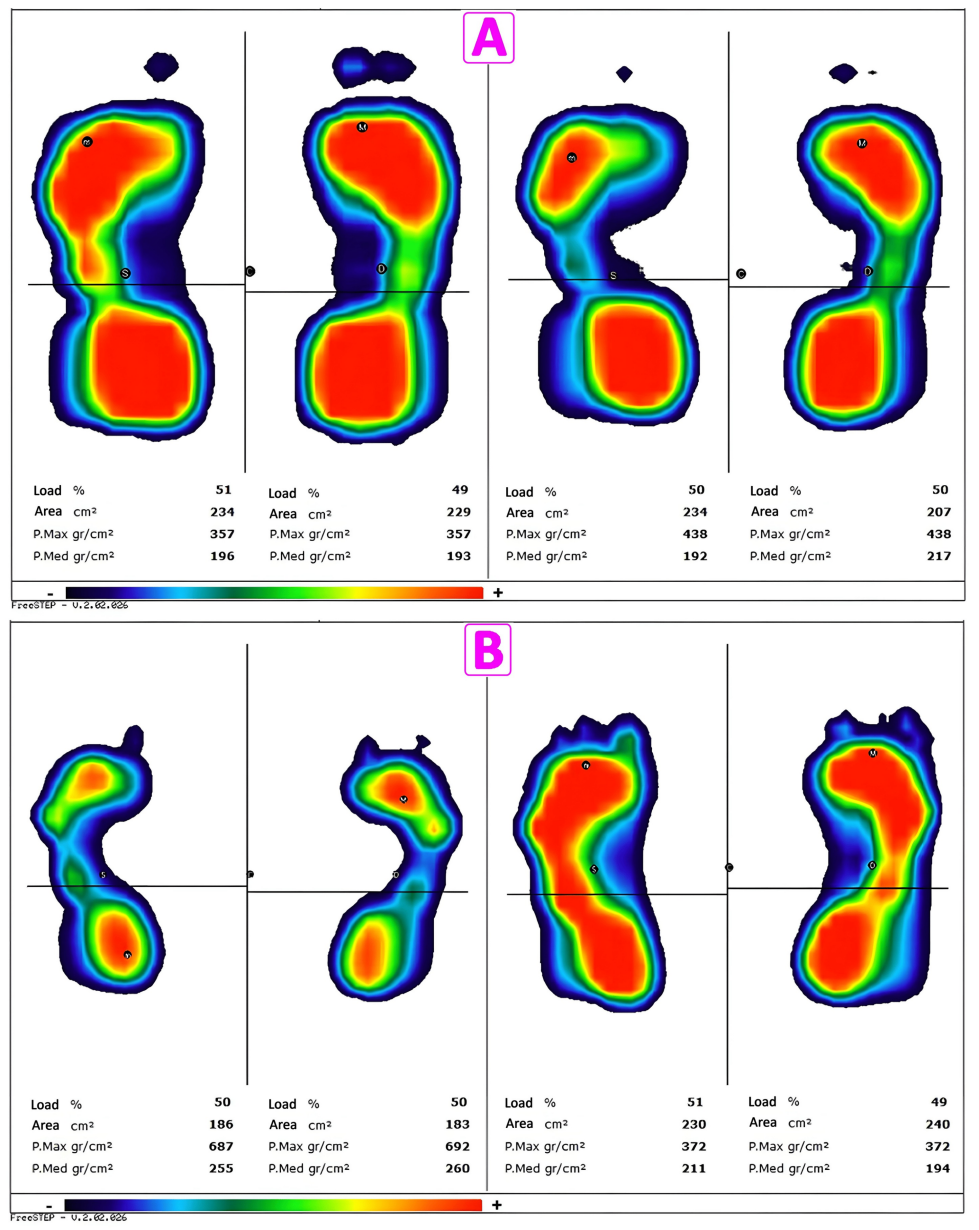
Stabilometric examination showing the change in plantar support A: Baropodometric examination on a patient who has had a tongue piercing for 16 years. B: Baropodometric examination on a patient with a nose piercing for six months. The two figures show the results of the stabilometric examination without (left) and with (right) piercing in two patients with tongue piercing for 16 years and nose piercing for six months, respectively. From the stabilometric image, the change in plantar support of the two subjects can be seen in more detail.

The results of the stabilometric analysis are displayed graphically in the form of a stabilogram, i.e., the displacement of the center of gravity as a function of time (Figures 6, 7). It is then possible to evaluate the strategies used by the subject to maintain static stability in an upright position, the position assumed by the subject, or abnormal swings or abrupt movements [12].

Examining the percentage of load on the platform tests of each patient (Table 5), we found variations in the podalic load of the left and right foot before and after piercing insertion. We saw changes in 20 out of 25 (80%) subjects. Of these, in nine out of 25, there was a decrease in the podalic load after piercing (36%); in 11 out of 25 (44%), there was an increase; in the remaining five (20%), there was no difference between before and after piercing. We also found that four out of 25 (16%) patients had a significant change in podalic load compared to the value of the other subjects, 20% and 8%, respectively.

**Table 5:** Stabilometric platform test results Details of patients who manifested changes in the podalic load of the left and right foot before and after piercing insertion.

Podalic load changes without/with piercing	Decreased podalic load without/with piercing	Increased podalic load without/with piercing
80% (20/25)	36% (9/25)	44% (11/25)

## Discussion

API method

For the clinical development of this work, we have used the Integrated Postural Analysis (API) method that the Italian Association for Postural Re-education (AIROP) has elaborated since 1996. In it, human posture is defined as the set of strategies implemented by the body following afferent stimuli present in the person (from the skin, viscera, teeth, eyes, spinal column, and feet, as well as biomechanical, psychological, and emotional nature) and manifested through dynamic neuro-myofascial, emotional, and biomechanical relationships [8].

AIROP establishes that a postural assessment is performed at various levels, starting with a general observation of the patient and progressively narrowing the field of analysis, until taking into consideration the body system involved or even the organ that may be primary in the postural dysfunction.

In order to be able to begin a postural assessment that will indicate the severity of the dysfunction and the presence of postural disharmonies both on a biomechanical and neurophysiological level, one must search for the area of primary dysfunction, which is a systemic and non-specific datum, evoked through the tests performed at the time of the examination. Then, it will be necessary to reduce and correct the adaptive compensations in progress. Finally, the degree of involvement of the different receptors and effectors of the “fine postural system” (FPS), where the somatic dysfunction has created an organ lesion, is determined in order to be able to complete a therapeutic program.

Posture and its dysfunctions

The current definition of posture is “the position assumed by the various parts of the body, in relation to each other and to the surrounding environment and the gravitational field reference system, in which there is absence of tension, absence of pain, and harmonious anatomical relationships” [13].

Thus, ideal posture means a condition in which all forces acting on the body are balanced and the body itself can perform all movements effectively and without pain [14].

The balance of muscular forces allows the body to maintain a stable and upright posture, correcting the shift of the center of gravity and recovering the upright position if it is lost. When this balance of forces is not optimal, the body is unbalanced, and postural dysfunction can occur. This can be caused by different factors, including breathing, swallowing, vision, pain in certain areas of the body, emotional factors, trauma or accidents, and nutrition, as well as inappropriate therapeutic devices such as glasses, corrective devices, orthodontic appliances, bites, or piercings [12].

The clinical diagnosis of postural dysfunction can be made by evaluating the skull, each body alignment, and the palpation of each muscle area in relation to an ideal standard posture [12,13].

The human body is a biomechanical system in which all functional posture units are interconnected. Consequently, small changes or incorrect postures of one of them often affect the other units, causing alterations or giving rise to postural compensations. In fact, any spatial or functional change in one posture unit causes changes in all segments belonging to the same sub-system. This creates re-compensation mechanisms that lead to adaptation by rotation and asymmetry of all functional units, and this may induce muscle pain and contractures, which can worsen with loss of length and alterations in muscle tone [9,13].

Postural Deficiency Syndrome (PDS)

If one considers poor posture as the result of the dysfunction of a single system, such as the oculomotor, vestibular, proprioceptive, podiatric, interceptive, or occlusal one, it becomes easier to explain the diversity of apparent symptoms, as this system can directly or indirectly interfere with various organs and apparatuses (e.g., the proprioceptive system). These symptoms cannot be explained by a conventional medical approach or placed in a clear and precise pathology of one or more specific organs: we are, therefore, in the so-called “functional” field.

All these signs and symptoms were first codified in the late 1980s by Dr. Da Cuhna, a physiatrist from Lisbon, who called it “postural deficiency syndrome” (PDS), and the resulting postural therapy was later systematized by Dr. Da Silva, an ophthalmologist from Lisbon [15,16]. They classified the signs and symptoms into two categories, major and secondary. The major functional signs of PDS are pain (headache, retro-ocular, chest or abdominal, arthralgia, and back pain), imbalance (with discomfort, nausea, dizziness, and unexplained falls), ophthalmological signs (asthenopia, blurred vision, diplopia, directional scotoma, and metatopsia), and proprioceptive signs (dysmetria, somatoagnosia, and body image assessment errors). The secondary functional signs of PDS could be articular (TMJ syndrome, stiff neck, lumbago, periarthritis, and sprain), neuromuscular (paresia and defect of motor control of extremities), neurovascular (paresthesia of the limbs and Raynaud’s phenomenon), cardiocirculatory (tachycardia and lipothymia), respiratory (dyspnea and fatigue), ENT (tinnitus and deafness), and psychic (dyslexia, dysgraphia, agoraphobia, orientation defect, concentration defect, memory loss, asthenia, anxiety, and depression).

In the early 1990s, Dr. Marino recoded the acronym PDS into “proprioceptive dysperception syndrome”; he developed a therapeutic protocol, initially thanks to the teachings of the aforementioned precursors, and then, since 2004, with the collaboration of a French ophthalmologist, Dr. Quercia, he has further explored this concept [17,18].

Based on the clinical results obtained, these two physicians highlighted other characteristics, for example, that modifications in muscle tone are only one aspect of other alterations caused by dysproprioception; that there is always an incorrect spatial localization of body districts; and finally, that the brain exhibits inability to precisely coordinate and integrate some sensory functions, such as sight and hearing, muscle tension, and movement.

Correlation Between Cranial Nerves and Posture

It is now important and interesting to remember which nerve endings are inevitably involved when a perforation is made in a part of the head, with the subsequent placement of a metal object in it. In general terms, we can say that a piercing in the area of the tragus or the auricle will involve the VII cranial nerve (facial and posterior auricular), the X cranial nerve (vagus and posterior auricular), the V cranial nerve (trigeminal and auriculotemporal), and the IX cranial nerve (glossopharyngeal); a piercing on the nose will affect the V cranial nerve (trigeminal), while one placed on the tongue will involve the XII cranial nerve (hypoglossal).

The part of the brain stem related to the cranial nerves is more complex than its counterpart in the spinal cord, as it governs more specific regulatory systems such as hearing, balance, and taste. The sensory and motor nuclei of the brain stem are similar to those of the spinal cord, and just as the neurons of the sensory and motor layers of the spinal cord are organized vertically, the neurons of the cranial nuclei also have a similar structure and tend to assemble and localize in characteristic areas at each level of the brain stem.

Thus, the spinal nucleus of the trigeminal nerve is a continuation of the dorsal horn of the spinal cord, and due to this anatomical arrangement, sensory fibers from the trigeminal and upper cervical nerves can ascend and descend through different segments; in addition, sensory information transmitted by the spinal and trigeminal nerves merge to form an uninterrupted map across the surface of the body [19].

The first studies on the relationship between the trigeminal nerve and the rest of the body began in the 1980s with experimental studies on animals. For example, Troiani et al. (1981) demonstrated that unilateral and bilateral transposition of the trigeminal nerve produced changes in the straightening of the head on the affected side [20].

Attention was then turned to humans, where Milam et al. confirmed that stimulation of the V nerve during dental treatment causes a parasympathetic discharge reflex and fainting similar to that caused by the vagus reflex [21]. Browne et al. showed a direct correlation between the trigeminal system and the cervical spine, in particular the masseter muscle, with activation of the head by and between mechanical stimulation [22]. A direct correlation was noted between mechanical stimulation of the masseter muscle and the electrical activity of the sternocleidomastoid muscle.

Cuccia and Caradonna ascertained a real correlation between the TMJ system and, therefore, the trigeminal system and body posture: they analyzed various factors that can influence “posture”, such as the emotional aspects of the person, the position of the neck and head, and the correct or incorrect oral function (breathing, swallowing, chewing, and structural anomalies), eye movements and visual systems, and the internal auditory system [23]. Furthermore, it has been seen that all these postural mechanisms are continuously adapting and are controlled by feedback and feed-forward responses [9].

Like the trigeminal nerve, the motor hypoglossal nerve has a postural value by innervating the musculature of the tongue and the regions above and under the hyoid bone with all the implications outlined above (as well as the importance it has by innervating these districts and from an osteopathic perspective), and they are both considered true body balancers together with the TMJ.

Furthermore, the hypoglossal nerve presents anastomoses not only with the trigeminal but also with the vagus and cervical plexus. On this subject, Cascio Rizzo et al. showed through a case report the correlations between ear piercings located at the crus of the helix, bilaterally (daith piercing), and the decreased intensity of chronic migraines: the stimulus induced by the piercing would inhibit nociceptive inputs by inducing the production of B-endorphins, being the area innervated precisely by the nerves mentioned above [24].

Piercings and postural instability

The study by Matheron and Kapoula hypothesized that “facial jewelry” or earlobe jewelry can interfere with somato-aesthetic signals guided by the trigeminal nerve, leading to postural problems such as eye misalignment, poor postural stability, and chronic non-specific back pain [7]. Four subjects with eyebrow, tragus, upper lip, and nose piercings who complained of chronic back pain, dizziness, headaches, and eye fatigue were evaluated. Following a medical examination and tests that ruled out any pathologies unrelated to the piercings, stability was assessed using a baropodometric platform. The tests were performed with and without piercings, with eyes open and eyes closed. When the piercings were removed, immediate improvements in postural stability were recorded, and at the three-week follow-up, all subjects reported a reduction in back pain or complete relief from pain, showing a significant improvement in postural stability.

Our clinical study on 25 subjects shows that afferent stimulation through piercings causes a disturbance, apparently transitory, on the fine postural system. The tests showed a significant overall change in the postural system of the subjects, in which worsening was partly manifested after the insertion of the “piercing perturbation” (22% of the cases); also, in 12.5% of the patients, there was improvement. There were no cases in which the system remained unchanged. This overall trend was found in all the tests with, however, not always overlapping characteristics, but with differentiated notes based on the location of the piercing. At the postural tone harmony (ATP) test, the tongue was the most sensitive, showing an improvement after reinsertion of the piercing in two (50%) patients. At the Barrè test in A-P projection, there were no significant changes, while it is interesting to note at the index finger test how the changes are more evident in the absence/presence of tongue piercings (41.7%); in patients with multiple piercings, we observed a change in both, of which worsening was observed in one (50%).

The data we collected from the tests performed highlight the following: the postural system does not always react in the same way to direct stimulation; responses are more evident where the nerve endings are more numerous; the greater the number of stimulations, the greater the response to the stimulus; and the same stimulus does not always give the same feedback for all study participants, and therefore, this must be in relation to the individual capacity of the proprioceptive system and its specific compensatory ability.

## Conclusions

Based on our analysis and the tests carried out on this sample of 25 patients, it is possible to state that piercings can cause various reactions, including postural alterations. The explanation is that these modifications are associated with the anomalous stimulation of the cranial nerves involved, producing various disturbances in the postural tonic system. However, not all the results are univocal, and it would seem that the postural alterations were better integrated by those who had had a piercing for a longer time, as if the system had considered the removal of the piercing itself as an “alteration”, and therefore, in a good number of subjects, the proprioceptive system was able to integrate the stimulus itself. A statistical limitation could be applied to our study, and it would also be advisable to carry out the tests following a greater absence of the perturbing external stimulus. However, this restriction could not be overcome, as the patients did not agree to the request to wait longer before relocating the piercing. Therefore, it is recommended to offer the tests to people who are planning to have one or more piercings placed and then to repeat them a few months after placement.

Regardless, the results obtained suggest the existence of a correlation, worthy of further investigation, especially due to the widespread nature of the phenomenon, which could constitute a public health problem. Despite the limitations mentioned, we can nevertheless affirm the existence of a well-founded link between postural alterations and the presence or removal of piercings. It is then clear that, in addition to what may be infections or other complications, the will to insert a “jewel” in one’s body can lead to changes that are not often manifestly attributable to the area of placement itself, since orofacial piercings seem to cause sensitive postural alterations that can be traced back to PDS. In conclusion, when a person decides to wear piercings, they should take into consideration the possible medical complications of these practices and the postural consequences that derive from them; however, their removal modifies the postural system and reintegrates basic proprioception.
